# A Pilot Qualitative Study to Better Understand the Factors Related to Suicides and Inform Public Health Action Across a Predominantly Coastal and Rural Area: Cornwall, Southwest of England

**DOI:** 10.3390/ijerph23010035

**Published:** 2025-12-25

**Authors:** Paula Chappell, Jane Horrell, Kerryn Husk, Beth Simons, Richard Alan Sharpe

**Affiliations:** 1 Wellbeing and Public Health Service, Cornwall Council, Truro TR1 3AY, UK; 2 Peninsula Medical School, Faculty of Health, University of Plymouth, Devon PL4 8AA, UK; 3 European Centre for Environment and Human Health, Department of Public Health and Sport Sciences, University of Exeter, Penryn TR10 8RD, UK; 4 Health Determinants Research Collaborative (HDRC), Public Health, Cornwall Council, Truro TR1 3AY, UK

**Keywords:** suicide, rural, coastal, surveillance, mental health, public health, pandemic

## Abstract

Background: Better understanding factors leading to suicide and prevention opportunities is a global public health priority. This qualitative pilot study tested whether reviewing inquest recordings could generate insights during COVID-19 and inform public health prevention programmes across a predominantly rural and coastal area where there are significant health inequalities. Methods: Fifty-five inquest recordings reached a suicide conclusion between March 2020 and January 2021. Stratified sampling was used to obtain two samples from each month. Template analysis was employed to thematically analyse data from 30 inquests. Results: Risk factors during this period were social isolation, anxiety, difficulty in routine creation and maintenance, low mood and economic impact. Remote working in a more rural/coastal area impacted both healthcare service users and staff. Lockdown and other multiple risk factors impacted those at increased risk of poor mental health and suicide. Conclusions: There is a need to identify those at risk and with deteriorating mental health. All age trauma-informed approaches are needed to prevent individuals from reaching crisis along with more equitable services and community support due to the complex nature of suicide. This requires consideration of digital access/exclusion, training, continuity of care and enhanced care of those with additional needs and multiple vulnerabilities.

## 1. Introduction

Risk of suicide is impacted by many social, relational, psychiatric and psychosocial factors [1]. These can include long-term conditions, particularly pain [2], financial insecurity [3], social isolation, drug and alcohol misuse, self-harm, a history of mental illness (e.g., a diagnosable mental health condition such as generalised anxiety disorder, mild/moderate depression or major depressive disorder) [4] and living in more coastal and rural areas [5]. While more rural areas across England are both remarkable and beautiful places, they have some of the highest rates of suicide. Typical risk factors in more rural areas include there being a higher burden of disease, seasonal and reduced employment opportunities, poor transport, increased distance to services, greater social isolation and impacts resulting from COVID-19 [5]. Gaining greater insights into complex overlapping risk factors is a public health priority because of the potential to inform more adaptive suicide programmes [6], which is the focus of this pilot qualitative study.

In England, the coroner’s office conducts publicly held inquests when the cause of death is unknown and aim to develop appropriate and verifiable information to determine the cause of death [7]. To our knowledge, no previous study has combined evidence from suicide surveillance systems (a digital record of all suicides) with coroner records during COVID-19 to determine whether this approach provides an opportunity to better inform suicide prevention efforts in a rural and coastal area of England. Using different information sources such as coroner inquest records in this context is a public health priority because the majority of individuals do not disclose when they are in crisis, potentially due to stigma, shame and a reluctance among people to discuss their problems with others [8,9]. Furthermore, undertaking a qualitative review of the data held by coroners provides an opportunity to gain more professional insights into the causes of suicide [10], which was a priority during COVID-19. This is of interest because coroner records hold more decedent characteristics than information collected by the police [11] and other services, which are often used in suicide surveillance systems.

Several international studies have adopted qualitative approaches using coroner records. These have helped to identify individuals who had not accessed health services due to a range of life events [12], as well as specific risk factors among different populations such as autistic individuals [13], older men (69%), women (50%) and those who did not have a known mental health condition prior to suicide [14]. Other methodologies that have been adopted include a mix of interviews, coroner records and linked health records to identify suicide risk factors and prevention opportunities [6,15], which was not possible in this study due to the restrictions of COVID-19. Despite this, using coroner records can help to better understand risk factors for suicide and enable the timely adaptations of prevention actions [16,17], particularly among more nuanced and rare cases of suicide [10].

Informing suicide prevention during COVID-19 was a priority because pandemics have previously led to increases in suicide-related outcomes such as suicide ideation and attempts [18]. Similarly, a systematic review reported a non-significant downward trend in suicides but a rise in suicide ideation and attempts during COVID-19 [19]. In contrast during COVID-19 there was no observed change in the global rate of suicide [20] in the first 9–15 months of the pandmic, with the exception of some areas where suicide rates were greater than expected [21]. Across Japan a decrease in suicides prior to COVID-19 and then an increase between 2020 and 2023 [22] was observed. In Mexico, there was an increase in suicide across all age groups during COVID-19 [23].

The psychological impact of lockdowns, fear of infection, frustration, insomnia and depression [24,25,26] are all additional risk factors. Furthermore, the collective quarantine measures have been linked to increased social isolation, risk of suicide and adverse mental health outcomes [24,27,28]. For these reasons, there was a need to better understand the needs and risks faced by a number of vulnerable population groups during the unique consequences of COVID-19 and national responses [29,30,31]. For example, during this period, inpatient and outpatient services were reduced to limit the risk of virus spread [32], which limited physical and perceived access to healthcare support.

Undertaking this study in Cornwall, South West of England was novel because it is a predominantly rural area that has a consistently significantly higher rate of suicide when compared to regional and national averages [33]. The county also has one of the largest coastlines across England and communities experience a range of health inequalities [5]. For example, over 60% of households live in deprivation [34], which, alongside rurality, is a known risk factor for suicide. To inform timely and adaptive suicide prevention, Cornwall has an established Public Health-led Real Time Suicide Surveillance system (RTSSs), which was developed to identify and respond to emerging risk factors. The RTSSs in Cornwall collects information on the timing, method and place of death from the police, which is used to inform the postvention intervention (i.e., support available for those affected by a suicide). The system also collects wider information collected from the National Health Service and the Local Authority.

A limitation of the RTSSs is that it does not include information disclosed during inquest hearings, hence the need for a different methodology during COVID-19. The use of inquest recordings during COVID-19 provided a way to better understand related suicide risks, which were needed to inform public health interventions. The coroner investigates all deaths due to unknown, violent or unnatural causes, which include suspected suicides [7]. This is achieved by reviewing evidence to determine whether each case reaches a suicide conclusion, regardless of who reports the death. The process is designed to establish who the deceased was and where, when and how they died. Inquests are held publicly and are a formal process. Unlike a criminal court case, there is no prosecution and defense. During the process, the Coroner collects relevant information from family members and statutory and non-statutory services [35]. In the UK, this does not include psychological autopsies, which are carried out during criminal investigations to identify the cause of death [36]. For this reason, this pilot qualitative study sought to understand the risk and protective factors for suicide and the specific impact of pandemics and test if reviewing inquest recordings during COVID-19 could help better inform timely Public Health (PH) suicide prevention programmes. This qualitative study aimed to review suicide inquest hearings held in 2020/21 to assess the following:

1. The impact of COVID-19 on health services and suicide;
2. Individual risk factors contributing to a suicide; and
3. Other contributing factors leading up to a suicide.

## 2. Methods

### 2.1. Study Development

This study was developed and implemented by the PH team, Cornwall Council (RAS & PC) and University of Plymouth (JH & KH). RAS and PC hold responsibility for the RTSSs and the public mental health and suicide prevention programme and have an existing relationship with the coroner. To prevent bias, the researcher (JH) undertook data analysis independently. The study was conducted to inform the prevention programme following the quality improvement framework [37] and in adherence to Cornwall Council’s Business Privacy Impact Assessment (BPIA), enabling ethical conduct and preventing the disclosure of identifiable information. Formal ethical approval was not required, as inquests are public hearings and the General Data Protection Regulation and the Data Protection Act no longer apply to identifiable data relating to a deceased person [38].

### 2.2. Population of Interest

The Senior Coroner granted permission to access inquest recordings for suicide deaths between March 2020 and January 2021 to help respond to the pandemic and subsequent restrictions [39]. Purposive sampling was used to identify recordings and then stratified sampling was applied to obtain two samples (one male, one female) from each month. A total of 113 suspected suicides were reported to the coroner during this period, 18 of which reached conclusions other than suicide. Of the 95 remaining, 62 reached a suicide conclusion and 33 were yet to complete the inquest process. Incomplete or untraceable recordings were excluded, providing an eligible sample of 55 recordings. Stratified sampling was combined with RTSSs data to obtain 30 cases for inclusion.

### 2.3. Linking Suicide Cases with RTSSs

Data on the decedent were obtained from inquest recordings and linked to RTSSs data for verification, demographic data and contributing factors. This offered additional insights, including pre-existing conditions, environmental factors and the systematic assessment of service use.

### 2.4. Qualitative Procedure/Analysis of Inquest Recordings

**Figure 1:**
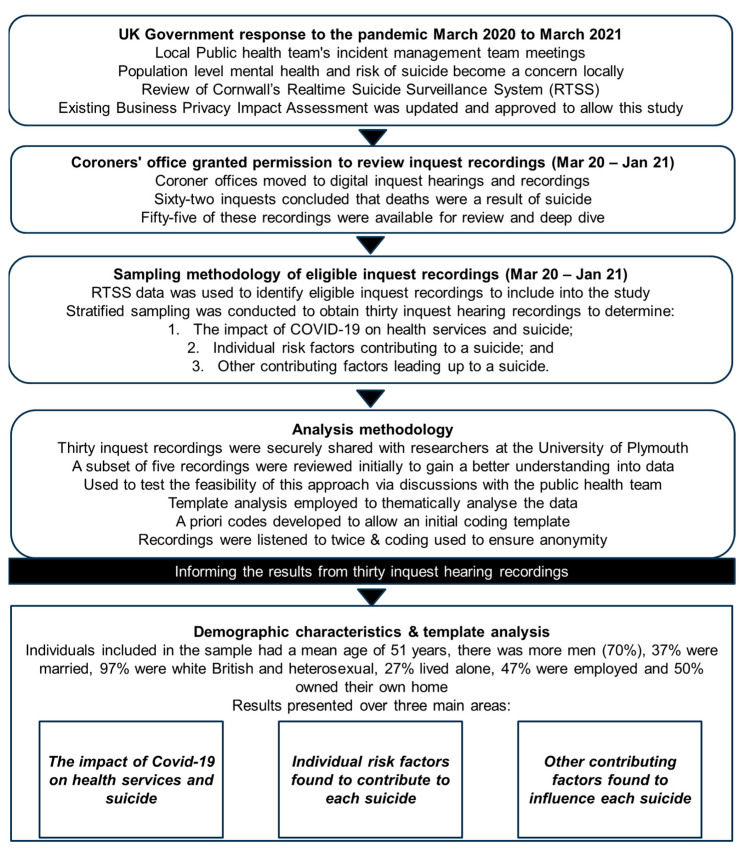
Overview of the deep dive into coroner inquest recordings where there was a conclusion of suicide, Cornwall, South west of England.

The analytical process (Figure 1) involved four key stages: (1) Familiarisation; (2) A priori (deductive) coding; (3) Inductive coding and (4) Refinement.

1. Familiarisation: A subset of recordings (n = 5) was reviewed by the researcher (JH) for familiarisation. Uncertainties were discussed and resolved with the Consultant in Public Health (RAS) and Advanced Public Health Practitioner (PC).
2. A priori coding using an Nvivo 12 Template Analysis approach [40] applying four themes: (i) Evidence informing understanding COVID-19 impacts; (ii) Service use; (iii) Contributory factors leading up to the suicide; and (iv) Experience of friends and families.
3. Inductive coding, including clustering of emerging themes and identification of hierarchical and integrative relationships.
4. Coding refinement aligned to the study aims.

A subset of ten inquest recordings from the 30 subjects were listened to twice and detailed field notes were made alongside the coding. The field notes were summarised and interrogated to develop analytic themes to synthesise findings and apply to the research questions (JH, PC and RAS). The template was iteratively refined as it was applied to the remaining data, with modifications made in response to newly identified material, culminating in a final template encompassing relevant data. Inquest recordings were numbered throughout the analysis process. Field notes and findings were discussed and developed with RAS and PC and the Cornwall Multi-Agency Suicide Prevention Group (MSPG). This group includes professionals and people with lived experience.

We have not provided a full summary of demographic characteristics or provided quotes from the recordings due to the small sample, risk of identification and sensitivity of suicide.

## 3. Results

The mean age of the sample was 51 years, with women ranging from 18 to 60 years (mean = 49) and men ranging from 29 to 90 years (mean = 52). A total of 70% of decedents were men, which was reflective of the proportion of suicide deaths among men and women in Cornwall. The majority of decedents were white British (97%) and heterosexual (97%). However, individuals’ living circumstances (27% lived alone), employment status (47% in full time employment) and housing status (50% homeowners) varied (Table 1). The recordings corresponded well to RTSSs data, supporting the reliability of the qualitative research.

Of the 30 included cases reviewed, people accessed services ranging from primary care to specialist secondary and acute National Health Services (NHS). A total of 37% of people accessed community mental health services and 27% accessed NHS Talking Therapies for support with a mental health condition such as generalised anxiety disorder (GAD), mild/moderate depression or major depressive disorder (MDD). A total of 67% had spoken to their General Practitioner (GP) in primary care about their mental health. A total of 27% had also accessed their GP for their physical health, and 27% received specialist services for their physical health from NHS services. Another six people (20%) accessed self-help or support within the voluntary sector (Table 2). However, 17% of people who died by suicide had no record of accessing any support from the NHS, community or self-help. In line with our aims, the inquest recordings were grouped into three key factors: the impact of COVID-19 on health services; individual factors impacted by the pandemic; and contributory factors leading up to suicide (including service use).

### 3.1. The Impact of COVID-19 on Health Services

COVID-19 impacted services in terms of personal experience and services offered/delivered. The restrictive impact of accessing services remotely for those who felt uncomfortable/unfamiliar with technology was a factor. Staff and patients experienced the impact of remote delivery, by way of obstructing therapeutic alliance building and gaining true judgements on how patients felt. Service efforts to balance duties of care and the risk of hospital-acquired infection (HAI) appeared to cause gaps in interagency/team communication and/or follow-up care.

1. Service Provision
  (1) The impact of remote working on treatment delivery; (2) The impact of the pandemic on treatment pathways and discharge processes.
  - The impact of remote working on treatment delivery
    - Therapeutic alliance
      Many mental health services moved from in-person to telephone appointments impacting treatment delivery and receipt. Practitioners described difficulty in understanding how a patient really felt {5, 13, 44} or engagement difficulties, hampering therapeutic alliance-building {38}.
    - Impact of self-isolation on delivery
      Although face-to-face delivery was re-introduced when restrictions eased, staff self-isolation continued to impact the ability to conduct appointments in person {36}.
  - The impact of the pandemic on treatment and discharge processes
    - Working practices
      The pandemic impacted working practices within inpatient settings due to a need to balance the risk of HAI against the least restrictive approach to care.
    - Discharge planning
      Discharge planning was impacted relating to balancing duties of care and the risk of HAI. Consideration was required for the risk of patients contracting the virus or bringing it onto the ward versus the risk of them returning home. Personal protective equipment (PPE) was constrained, with no face masks or testing kits at this time. Under normal circumstances, the discharge policy contains two procedures: (1) where possible, family should be involved; (2) an assessment of need for follow-up care. In one example, the family were not involved {21} and a lack of testing capacity meant it was felt admission was not preferable.
      Consideration was required for discharging a patient into the care of a frail elderly relative who would be considered high risk upon virus contraction. The team would ordinarily provide enriched or additional support when discharging into the care of an elderly relative; this was curtailed due to restrictions {22}.
    - Referral pathways
      Discharge planning was impacted by delays in referral pathways. One person waited two weeks for a GP referral to mental health services, five days for urgent referral triage, and a further week for an appointment; it was during this time they took their life {22}. A transfer between teams failed to occur for another person, meaning they were ‘lost’ between teams {38}.
2. Access to services
  - The impact of the pandemic on accessing treatment
    - Access to medication
      Changes in GP surgery resulted in unsatisfactory access to antidepressant medication. In one case, a consultation with the new surgery was conducted by telephone, resulting in half the medication dose prescribed {44}. In another case, an increase in medication was agreed upon but delayed {54}.
    - Concerns around accessing support/treatment remotely
      Many of the deceased experienced a change from face-to-face service to telephone consultations {5, 12, 13, 36, 38, 44, 54}. Families felt face-to-face contact may have had a positive impact on care {36}. Some people withdrew from treatment with the intention of re-engaging once restrictions were lifted {38}. Others felt distrustful of technology and resisted the option of communicating remotely {54}.
  - Health anxiety
    Families/friends reported increased anxiety centred on the virus, including reluctance to access care due to fear of catching the virus {50}. Another case centred on fears of the deceased themselves having COVID, subsequently placing themselves in isolation and missing a GP appointment {9}.
    Some people experienced confines in accessing consultations and tests/test results for physical illness. In one case, the deceased lived with chronic pain but was informed an appointment to discuss surgery needed to wait until after the pandemic stabilised {8}. A further inquest detailed the deceased attending A&E over 20 times in two months and struggling to access diagnostic tests and results {51}.

### 3.2. Individual Factors Impacted by the Pandemic

There were a range of individual factors including social isolation, increased anxiety, difficulty in creating/maintaining routine, low mood and the economic impact:

1. Isolation

Isolation due to the pandemic was mentioned in general terms {3, 7, 36, 55} and in terms of a lack of visits to care homes {18} and hospitals {54}. References were made to isolation from friends and work {24, 32, 34, 36}.

2. Increased anxiety

Increased anxiety was mentioned in several ways. For some, restrictions intensified an underlying tendency for anxiety, with social distancing and working remotely causing general agitation {14, 49}. Families/friends reported sleep disturbances related to the pandemic {36, 54}. Others worried about how long the pandemic would last {7} and for family members who were frontline workers/clinically vulnerable {7}. Concerns lockdown restrictions would impact health were referenced by one family member {5}.

3. Difficulty in creating/maintaining routine

Some of the deceased were reported to have found the lockdown difficult to cope with due to the lack of routine. This was related to establishing a routine {24}, and loss of the previous one {17, 32, 36, 52, 55}.

4. Low mood

Restrictions appeared to impact motivation and lack of interest in family/friends/work {28, 45}, as well as general difficulties in adaptation to lockdown {17, 18, 36}.

5. Economic impact

One participant was reported to feel pressured over job insecurity and associated financial impacts {28}.

### 3.3. Contributory Factors

Qualitative analyses revealed a number of health-related contributory factors, which included both mental and physical health, cognition or memory loss and drug and alcohol use. Poor mental health was the most prevalent (Table 3). Most women (N = 8) were being treated for mental illness at the time of death. Nine men (43%) did not have, or never had, a mental health diagnosis. Those with a current diagnosis (N = 8) also had a history of mental illness. Two others had a historical diagnosis but were not receiving treatment at the time of death. Five men (24%) and three women (33%) had physical health conditions. Due to the pressures of COVID-19 on local healthcare systems (e.g., the National Health Service and the Local Authority), it was important to identify those who were previously in contact with services. GPs were the most commonly accessed service for mental health support, followed by community mental health services. Women were more likely to be involved with multiple services than men, and involvement with the criminal justice system featured in the histories of three people.

Other individual risk factors identified from qualitative syntheses included a range of environmental factors (Table 4). Environmental factors linked to the suicide cases included having access to lethal means, prolonged stress including having difficult relationships and experiencing stressful life events such as a relationship breakdown, bereavement and financial difficulties. Having a history of previous lifestyle and health factors was also present in the suicide cases reviewed (Table 5). Suicide attempts were present for seven males (33%) and six females (67%). Mental health diagnoses were present for eight women (89%), whereas nine men (43%) had no known history of mental health conditions. Childhood neglect/trauma was only represented in the male sample (19%). Importantly, for suicide prevention efforts, 42.9% of men had no history of a mental health condition prior to dying by suicide.

## 4. Discussion

**Figure 2:**
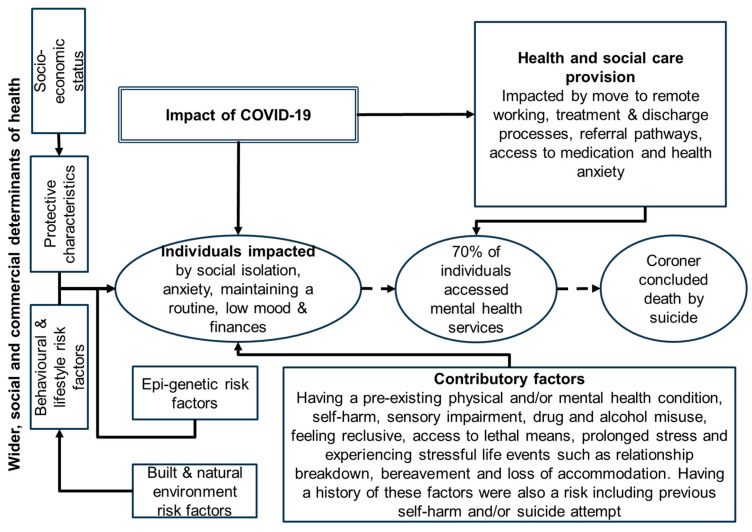
Concept model of factors contributing to suicides.

This novel qualitative study aimed to understand the risk and protective factors for suicide during COVID-19 and test if reviewing inquest recordings during this period provided a way to inform suicide prevention interventions. When compared to the real-time suicide surveillance system in Cornwall, the use of inquest recordings provided a deeper understanding of factors contributing to suicide during COVID-19 (Figure 2). This included a range of wider determinants of health that impact the risk of suicide through the provision of health and social care, individual, environmental and historical factors. Our findings show that these were exacerbated by COVID-19 (Figure 2) across a predominantly rural and coastal area that experienced a higher number of suicides compared to other more urban areas across England [5]. For example, COVID-19 impacted local health services (e.g., those delivered by the NHS) and limited access to support. Other factors included pre-existing mental health and/or physical health conditions, stressful life events and a history of self-harm. This further supports the need for parity of esteem between physical and mental health given the impact of poor physical health on mental health. A total of 37% of those who died by suicide were known to community mental health services. Twenty percent of cases reviewed had accessed acute or hospital psychiatry services, but most were known to primary care in the community (67%). This highlights the role of both mental health and community services in preventing the risk of suicide. However, 17% of people had no record of accessing NHS services, those in the community or self-help, which supports the need for wider prevention programmes than those focusing on service use.

Using inquest recordings provides a methodology for gathering insights into suicides during COVID-19, which are not routinely captured by surveillance systems. The experiences of friends and families added a richer understanding of the factors leading to suicide. Family and friends talked most often of experiences of isolation, increased anxiety, low mood and difficulties in creating and maintaining routine. Future approaches should include findings from Serious Incident Reviews in respect of patients accessing NHS-funded services in England. Despite this potential, inquest hearings are not routinely used to inform suicide prevention programmes, possibly because of limitations due to access and the resources required. Our study demonstrated that this approach could be routinely applied, particularly with the emergence of technology such as Artificial Intelligence (AI) [10].

### 4.1. What Is Already Known About This Topic?

#### 4.1.1. Health and Social Care Provision

COVID-19 had a profound impact on the delivery of healthcare services, with a rapid shift to remote working, difficulties in referrals, service provision and access to medications, which featured in the cases reviewed. This is consistent with a social prescribing study conducted during COVID-19. This reported that a shift to remote services to protect service users and staff resulted in a drop in referrals, presenting key challenges in adopting new ways of working [41]. In addition, those requiring mental health support [42] and those accessing and being discharged from services [43,44] were at increased risk. Better integration between community mental health and education, with early prevention including self-harm and suicide ideation, is needed [45] in both non-clinical and clinical settings [46].

#### 4.1.2. Individual Factors Contributing to Suicides

The use of inquest recordings enabled the identification of nuanced factors contributing to the risk of suicide, which included social isolation, increased anxiety, loss of routine as a result of the pandemic, low mood and economic impacts. This study supports the use of inquest recordings as a way to examine risk factors and the development of prevention actions, which is consistent with Cheung, Merry [47]. Additionally, our study found that risk factors included employment status and drugs and/or alcohol use, which is consistent with other findings [4,14]. Although national lockdowns posed a risk to some, it offered protection to others in the form of increased social support from families, friends and community networks. This may also have included reduced access to some suicide methods [48]. In contrast, for others, the lifting of restrictions led to increased anxiety related to re-engaging with activities/routines paused during lockdown. Also consistent with our findings, having a physical health condition [49] and determinants such as adverse childhood experiences, debt and drug/alcohol use disorders [50], domestic abuse [51] gambling [52] and financial/food insecurity [10] are all risk factors. Our findings identified risk factors consistent with other evidence, including low social support/isolation, physical and mental health needs, sleep disturbances, quarantine and exhaustion in the workforce [53]. Employment status was found to be an important factor, particularly in sectors such as construction, farming [54] and healthcare [55].

#### 4.1.3. Contributing Factors

These need to be considered alongside other contributory factors. The review of recordings highlighted that mental and physical health conditions were prevalent in records, which is considered in relation to other findings where individuals had a diagnosis of depression (33%), depressive/mood symptoms (47%) and physical health problems (55%) [47]. We also found that a range of environmental factors were highlighted, which included access to lethal means, prolonged stress and stressful life events such as relationship difficulties and bereavement. Other research has reported that relationship breakdowns were a factor for both women and men but are known to increase suicide risk more significantly in men [56], and bereavement remains a risk for mental health and suicide [57]. These findings need to be considered alongside co-existing physical health conditions and the described reduced access to healthcare [58].

Historical health and life factors were also dominant in the records reviewed, which included, for example, childhood trauma, prior depression diagnosis and previous self-harm and/or suicide attempt. Having a history of mental illness, self-harm and access to mental health services was an important factor consistent with other literature [4,59]. This is important to consider in prevention programmes because having a history of self-harm remains a lifetime risk [60]. Also, the risk of transition from suicidal ideation/thoughts to an attempt is moderate to high (up to 37%), particularly in those with a mental health disorder [61,62]. We also found that neurodivergence or sensory impairment was a factor, which aligns with previous findings [13] that autistic traits were over-represented in those who have died by suicide.

### 4.2. What Does This Study Add?

Inquest hearings offer a valuable adjunct to traditional approaches for understanding the factors surrounding suicide. Consistent with research [6], we found this methodology provided a novel way to make recommendations to improve suicide prevention programmes and should be more widely adopted. Linking this dataset with healthcare records, as well as discussing with professionals and friends/family, provides a further means to understand the needs of those who have not accessed services [12,16]. This is important because of the high proportion of individuals not known to mental health services, as well as other lifestyle risk factors such as in those bereaved by suicide [63].

Our study provides good evidence to support the use of inquest recordings alongside RTSSs. This supports a global need for improvements in data with implementation RTSSs to inform suicide prevention [64]. Using inquest findings routinely to identify emerging risk factors will help to inform current and future prevention programmes and may provide a means to improve RTSSs across England and internationally, including in detecting clusters [65]. This is important to consider because of insufficient evidence to support the use of clinical and pharmacological epidemiological surveillance [66].

The findings identified barriers pertinent to service provision, particularly in how they can best be designed to support, encourage and maintain engagement in support/treatment services. The individual cases reviewed experienced a complex blend of stressors that were not able to be met by one organisation/service alone. Prevention and clinical services should be developed to support people with multiple vulnerabilities such as those across mental health as well as drug and alcohol use, financial and housing difficulties to mitigate against future macro-level effects. In addition, there are a number of improvements, which include workforce and whole population training; continuity of care; early intervention; formal care arrangements; plans to respond to major events; support for those experiencing adverse childhood experiences; measures for those with sensory impairment; and equitable access to a range of services that can help address the multiple and overlapping risk factors for suicide [10]. Approaches must take a whole system and trauma-informed approach [67,68].

This should be developed alongside screening in primary care [69] and effective treatment for the prevention and management of suicide [70] through brief contact interventions in healthcare settings [71]. This could be achieved through improved training in primary care and the use of electronic care records [72]. These findings support the need to better understand the effectiveness of suicide prevention initiatives, with evidence supporting removing access to means [73] and a range of preventative programmes across the life course to be effective [74,75].

## 5. Implications of the Study for Current Policy and Practice

This study supports the need for local surveillance systems to adopt retrospective analyses of suicide cases alongside real-time monitoring. To be effective in suicide prevention, there needs to be improved data interoperability including improved data management and ethical consideration [76] that includes the use of coroner records. Other studies investigating the use of qualitative analyses alongside the use of AI highlights the need for a greater focus on multimodal data, which includes its availability/privacy, data abstraction and approaches used to identify prevention opportunities [10]. This approach provides an opportunity to improve prevention efforts by identifying new insights and novel risk factors [77]. Extending the findings of this study to support new novel approaches such as the use of retrospective analyses of coroner records or similar records provides an opportunity to identify timely, tailored and personalised prevention initiatives, which could consist of universal, selective and indicated interventions [78,79]. Future studies should also further explore the role qualitative analyses alongside the use of AI technologies to identify more cost effective approaches [80].

## 6. Strengths of This Study

The strengths of this study included access to an established RTSSs and novel access to inquest recordings held during COVID-19. It was informed by working with experts in RTSSs and suicide prevention, including a multi-agency suicide prevention group. The methodology provided an in-depth insight into the factors leading to suicides during COVID-19; however, it was not possible to review all deaths due to available resources. Our sampling process identified more men, which is consistent with national data and adds confidence to the adopted strategy [33]. A further strength was this study’s role in informing the response to COVID-19 across Cornwall. This provided a ‘real world’ example of translating evidence into practice because it supported the development of a novel community-based and multi-agency suicide prevention programme that aimed to tackle the identified risk factors.

## 7. Limitations of This Study

Some limitations exist. It was impossible to assess and compare risk factors prior to COVID-19 because of the restrictions in place. However, we compared the findings with an existing RTSSs and the shift to and availability of inquest recordings provided a novel opportunity to test whether this approach provided new insights. This does mean that future research would benefit from a comparison of inquest recordings prior to and after COVID-19. This work may not be fully representative of suicides during this time, although taking a sampling approach helped mitigate this despite the small sample size. By definition, this research could not include first-hand experience, so it relied upon interpretation by families, friends and professionals. Future work should engage those at risk of suicide, as well as develop AI and behavioural science to better understand suicide in a timelier manner [35]. The combination of these approaches will help develop global policy and practice related to the wider determinants of health and suicide prevention.

## 8. Conclusions

This study provides new insights into the factors that contribute to deaths by suicide in a predominantly coastal and rural area of England, particularly those that take place during a global pandemic characterised by lockdown restrictions. Using this methodology provides a means to understand risk factors not readily reported in RTTSs or healthcare records to help inform suicide prevention programmes. It provides an opportunity to prepare for future macro-level events and could be used routinely to inform policy and practice. Evidence suggests that new models are required that lessen the gap between individual need and the demand made for service provision. Future suicide prevention measures should be trauma-informed across the life-course and take a whole system approach to mitigate the impact of wider determinants on mental health and suicide.

## 9. Declarations

The writing-up of this study has been delivered through the National Institute for Health and Care Research (NIHR) [HDRC Cornwall]. The views expressed are those of the authors and not necessarily those of Public Health, Cornwall Council, the NIHR or the Department of Health and Social Care.

This report is independent research supported by the National Institute for Health Research Applied Research Collaboration South West Peninsula. The views expressed in this publication are those of the authors and not necessarily those of the National Institute for Health Research or the Department of Health and Social Care.

## Figures and Tables

**Table 1:** Demographic characteristics obtained from the RTSSs.

Demographic Characteristics		%	Mean	SD
Age			51	18.2
Gender				
Male		<70%		
Female		<30%		
Marital status				
Single		<40%		
Married		<37%		
Divorced		<17%		
Widowed		<7%		
Ethnicity				
White British		<97%		
White (other)		<3%		
Sexual Orientation				
Heterosexual		<97%		
Homosexual		<3%		
Unknown		0%		
Living situation at time of death				
Alone		<27%		
Spouse/partner		<40%		
Parents		<20%		
Adults (non-family)		<7%		
Other family		<7%		
Occupation at time of death				
Employed (full-time)		<43%		
Employed (part time)		<3%		
Student (full-time)		<3%		
Unemployed		<20%		
Long-term sick/disabled		<3%		
Retired		<27%		
Housing status at time of death				
Owner occupier		<50%		
Council/housing association		<3%		
Other-with family		<17%		
Other-with friend		<3%		
Other (family-owned house)		<3%		
Care provider		<3%		
Homeless/no fixed abode		<3%		
Holiday let		<3%		
Unknown		<13%		

Number of cases reviewed and less than <% have been used to prevent the potential disclosure of individuals.

**Table 2:** Review of access to services among those dying by suicide from coroner inquest recordings.

#	Case ID	Mental Health						Physical Health			3rd Sector				Total
		Mental Health Services	Primary Care	Talking Therapies	Acute or Hospital Psychiatry	Detained Under Mental Health Act	Voluntary Hospital Admission	Primary Care	Specialist Support tests	Specialist Support	Substance Use Support	Bereavement Support Services	Community Support	Self-Help	
1	3		1	1						1			1		4
2	5	1	1	1						1					4
3	7														**0**
4	8	1	1		1		1			1	1				6
5	9							1							1
6	12	1	1	1							1				4
7	13		1					1	1						3
8	14	1	1		1				1						4
9	16									1					1
10	17														**0**
11	18														**0**
12	21	1				1									2
13	22	1	1		1		1	1							5
14	24		1	1	1										3
15	25		1							1	1				3
16	28		1	1											2
17	31									1					1
18	33	1	1	1								1	1		5
19	35	1	1												2
20	37	1	1		1										3
21	40		1	1				1		1					4
22	43		1	1									1		3
23	44		1												1
24	49														**0**
25	50							1							1
26	51		1					1	1						3
27	52														**0**
28	53	1	1					1	1		1				5
29	54	1	1		1					1					4
30	55		1					1				1		1	4
**Total**		**11**	**20**	**8**	**6**	**1**	**2**	**8**	**4**	**8**	**4**	**2**	**3**	**1**	78
**%**		**37%**	**67%**	**27%**	**20%**	**3%**	**7%**	**27%**	**13%**	**27%**	**13%**	**7%**	**10%**	**3%**	-

**Table 3:** Individual themes arising from reviewing the health status of those who died by suicide from coroner inquest recordings.

Health *	Male% (n = 21)	Female% (n = 9)
Mental health conditions (known to services including GP)		
Depression	38.1 (8)	66.7 (6)
PTSD	4.8 (1)	
Personality disorder	4.8 (1)	11.1 (1)
Psychotic episode	9.5 (2)	
Bipolar disorder		11.1 (1)
Heightened anxiety	4.8 (1)	11.1 (1)
Addictions	14.3 (3)	22.2
Not known to mental health services at time of death	52.4 (11)	11.1 (1)
Physical health conditions		
Living with chronic pain	4.8 (1)	11.1 (1)
Concerns for health	33.3 (7)	11.1 (1)
Reluctance to engage with medical services	9.5 (2)	
Physical illness	23.8 (5)	33.3 (3)
Menopause		11.1 (1)
Cognition		
Experiencing problems with memory	4.8 (1)	
Experiencing confusion/losing touch with reality	4.8 (1)	
Mild cognitive impairment	4.8 (1)	
Low confidence	4.8 (1)	11.1 (1)
Feeling a burden	4.8 (1)	
Becoming increasingly withdrawn prior to death	9.5 (2)	
Lifestyle		
Alcohol/drug use	23.8 (5)	22.2 (2)
Reclusive way of life	4.8 (1)	

* Individuals may be attributable to more than one category.

**Table 4:** Individual themes arising from reviewing environmental factors contributing to suicides from coroner inquest recordings.

Environmental	Male% (n = 21)	Female% (n = 9)
Access to lethal means		
Prescription drugs	9.5 (2)	33.3 (3)
Illegal drugs	9.5 (2)	
Prolonged stress		
Loneliness	4.8 (1)	
Difficulty sleeping	9.5 (2)	11.1 (1)
Caring responsibilities	4.8 (1)	
Difficult family/friend relationships (including social media)	19 (4)	33.3 (3)
Stressful life events		
Relationship breakdown	19 (4)	33.3 (3)
Loss of child (child removal or death)	4.8 (1)	
Loss of job/business (not COVID-19-related)	4.8 (1)	11.1 (1)
Coming out as gay	4.8 (1)	
Bereavement	14.3 (3)	44.4 (4)
Financial concerns	19 (4)	11.1 (1)
Loss of accommodation	9.5 (2)	
Family illness		11.1 (1)
Exposure to another person’s suicide (not family)		22.2 (2)

Individuals may be attributable to more than one category.

**Table 5:** Individual themes arising from reviewing historical lifestyle and health factors contributing to suicides from coroner inquest recordings.

Historical	Male% (n = 21)	Female% (n = 9)
Previous suicide attempts/history of self-harm	33.3 (7)	66.7 (6)
Family history of suicide	4.8 (1)	
History of mental health conditions		
Anorexia		11.1 (1)
Depression	47.6 (10)	66.7 (6)
Personality disorder	4.8 (1)	11.1 (1)
PTSD	4.8 (1)	
Bipolar disorder		11.1 (1)
No history of mental health conditions	42.9 (9)	
Childhood abuse, neglect or trauma	19 (4)	
Sensory conditions	9.5 (2)	
Experiencing abuse/displaying abusive behaviours	9.5 (2)	

Individuals may be attributable to more than one category.

## Data Availability

The data presented in this study are available on request from the corresponding author subject to approval from the Coroner’s Office, Cornwall Council. This is required due to the sensitivity of the information and ethical considerations.
